# Clinical experience with the AKT1 inhibitor miransertib in two children with PIK3CA-related overgrowth syndrome

**DOI:** 10.1186/s13023-021-01745-0

**Published:** 2021-02-27

**Authors:** Karina Forde, Nicoletta Resta, Carlotta Ranieri, David Rea, Olga Kubassova, Mark Hinton, Katrina A. Andrews, Robert Semple, Alan D. Irvine, Veronika Dvorakova

**Affiliations:** 1 Dermatology, Children’s Health Ireland at Crumlin, Dublin 12, Ireland; 2 Department of Biomedical Sciences and Human Oncology (DIMO), Division of Medical Genetics, University of Bari “Aldo Moro”, 70124 Bari, Italy; 3 Clinical Imaging, Children’s Health Ireland at Crumlin, Dublin 12, Ireland; 4 Image Group Analysis (IAG), London, UK; 5 University of Cambridge Metabolic Research Laboratories, Wellcome Trust-MRC Institute of Metabolic Science, Addenbrooke’s Hospital, Cambridge, UK; 6 Department of Clinical Genetics, Cambridge University Hospital NHS Foundation Trust, Cambridge, UK; 7 Centre for Cardiovascular Science, University of Edinburgh, Edinburgh, UK; 8 Clinical Medicine, Trinity College Dublin, Dublin, Ireland

**Keywords:** PIK3CA-related overgrowth spectrum (PROS), Miransertib, AKT inhibitor, Next generation sequencing (NGS), Targeted treatment

## Abstract

**Background:**

PIK3CA-related overgrowth spectrum (PROS) refers to a group of rare disorders, caused by somatic activating mutations in PIK3CA, resulting in abnormal PI3K-AKT-mTOR pathway signalling. Significant associated morbidity is frequently observed, and approved treatments are lacking. Miransertib (ARQ 092) is a novel, orally available, selective pan-AKT inhibitor with proven in vitro efficacy. Following recent results of the use of AKT inhibitors in Proteus syndrome (PS) and AKT-mutant cancers, we investigated its therapeutic use in two patients with severe PROS who had exhausted conventional treatment methods.

**Results:**

Two patients, one with CLOVES variant (P1) and one with facial infiltrating lipomatosis and hemimegalencephaly (P2), were commenced on miransertib treatment on a compassionate use basis. In patient one, intra-abdominal and paraspinal overgrowth had resulted in respiratory compromise, obstructive uropathy, dysfunctional seating and lying postures, and chronic pain. In patient two, hemifacial overgrowth and hemimegalencephaly had caused difficulties with articulation and oral function, and refractory epilepsy. Miransertib treatment was continued for a median duration of 22 months (range 22–28). In patient one, alleviation of respiratory compromise was observed and functionally, seating and lying postures improved. Serial volumetric MRI analysis revealed 15% reduction in calculated volumes of fatty overgrowth between treatment commencement and end. In patient two, reduction in seizure burden and improved parent-reported quality of life measures were reported. Treatment was discontinued in both patients due to lack of sustained response, and poor compliance in year two of treatment (P2). No significant toxicities were reported.

**Conclusion:**

We report the first paediatric case series of the use of miransertib in two children with PROS. Objective clinical response was observed in patient one, and improvement in key qualitative outcomes was reported in patient two. Treatment was well tolerated with no significant toxicities reported. This case series highlights the potential therapeutic utility of miransertib in selected paediatric patients with severe PROS, and further demonstrates the potential for re-purposing targeted therapies for the treatment of rare diseases. An open label, Phase 1/2 study of miransertib in children with PROS and PS is underway to more accurately assess the efficacy of miransertib in the treatment of PROS disorder (NCT03094832).

## Background

PIK3CA-related overgrowth spectrum (PROS) refers to a group of heterogeneous, rare disorders characterised by segmental progressive overgrowth, caused by somatic activating mutations in phosphatidylinositol-4 5-bisphosphate 3-kinase catalytic subunit alpha (PIK3CA) gene [1]. The PI3K/AKT/mTOR pathway is essential for normal cellular functions including growth, metabolism, and proliferation. PIK3CA encodes the p110alpha subunit of phosphoinositide 3-kinase that converts phosphatidylinositol 3,4-bisphosphate to phosphatidylinositol 3,4,5-triphosphate. Subsequent activation of AKT and mTOR signalling results in promotion of tissue growth. Dysregulation causing over-activation of the pathway results in increased cellular metabolism, angiogenesis and reduction in cellular apoptosis [2].

In recent years, next generation sequencing (NGS) has significantly expanded the understanding of the molecular aetiology of overgrowth syndromes [1, 3–6]. In 2014, the identification of PIK3CA mutations in a group of patients with heterogeneous, but overlapping, clinical features [7] resulted in the subsequent development of diagnostic criteria for the newly termed PIK3CA-related overgrowth spectrum (PROS) [1]. These disorders are extremely rare, although the exact prevalence is difficult to estimate owing to the phenotypic heterogeneity of disorders and variability of correct clinical diagnoses to date [8].

PIK3CA-related overgrowth spectrum (PROS) disorders are associated with significant morbidity and mortality. Past therapeutic approaches centred around symptomatic surgical or medical management of associated complications, with minimal success. There are no approved targeted medical treatments.

The P13K/AKT/mTOR pathway is recognised as one of the most frequently dysregulated pathways in human cancers, with PIK3CA identified as the most commonly mutated proto-oncogene in somatic cancer [9]. With the advent of increasing knowledge regarding the oncogenic implications associated with dysregulation of the P13K/AKT/mTOR pathway, the development of several small molecules targeting different key components of the pathway have come under clinical investigation. Thus, opportunities for targeted treatment of overgrowth disorders are now being recognised.

The mTOR inhibitor sirolimus has been used with partial success in overgrowth disorders. A previous open label multicentre study reported a modest mean reduction in total tissue volume at affected sites (7.2%) in 23/30 patients who were evaluable after 26 weeks treatment. Treatment was associated with a high rate of adverse events, many severe, resulting in consequent treatment withdrawal in a significant number (7/39; 18%) [10]. In addition, a previous study of its use in children with heterogeneous complex vascular disorders appeared to show greatest response rates in those with lymphatic malformations [11]. Furthermore, since sirolimus fails to effectively inhibit mTORC2, feedback activation of PI3K and AKT persists, rendering it a less attractive option in the long-term management of patients with PROS. The focus for future therapeutic options has expanded to include AKT inhibitors, PI3K inhibitors and dual PI3K-mTOR inhibitors, many of which are undergoing investigation in oncology trials [12]. More recently, Venot et al. reported a substantial clinical response in 19 patientswith PROS treated with the PI3K inhibitor alpelisib (BYL719), in an open label, non-RCT case series, along with a favourable safety profile [9], further supporting the repurposing of agents targeting the P13K/AKT/mTOR in the treatment of PROS.

Miransertib (ARQ 092) is a novel, orally available, highly selective pan-AKT inhibitor with proven in vitro efficacy [13]. Lindhurst et al.previously identified a reduction in AKT phosphorylation and downstream targets in cells of patients with Proteus syndrome treated with miransertib [14]. A subsequent phase 0/1 study of miransertib in six patients with Proteus syndrome showed a 50% reduction in pAKT phosphorylation in affected tissues in 5/6 participants four months after treatment initiation [15]. Subsequently, Leoni et al. reported the successful treatment of a patient with Proteus syndrome and relapsed low-grade serous ovarian carcinoma with miransertib, who demonstrated partial response on computed tomography (CT) imaging at 24 weeks, and achieved complete remission after 22 months of treatment, along with significant reported improvement in quality of life scores [16].

## Methods

### Aims

To investigate the therapeutic use of pan-AKT inhibitor miransertib (ARQ 092) in two paediatric patients with severe PIK3CA-related overgrowth spectrum disorder, both of whom had exhausted conventional treatment methods.

### Participant characteristics

#### Patient one

Patient one was a 16 years old female with Congenital Lipomatous Overgrowth, Vascular malformations, Epidermal nevi, Scoliosis/Skeletal/Spinal anomalies (CLOVES) phenotype [17, 18]. She was born to non-consanguineous parents with a birth weight of 2.89 kg (50th centile) and occipitofrontal circumference of 31.6 cm (10th centile) and was noted to have a large posterior thoracic wall lipoma and large feet with splayed toes at birth. Subsequent musculoskeletal examination identified a dislocated right hip and bilateral talipes equinovarus. Progressive intra-abdominal and paraspinal lipomatous overgrowth created significant difficulties with mobility and seating; these were treated with debulking procedures and liposuction during the first 10 years of life. While these procedures lead to partial but temporary improvement, the lipomatous lesions regrew within 12 months. Despite repeated orthopaedic intervention, she developed progressive lower limb discrepancy and subsequent flexed deformity of the right lower limb, resulting in loss of function.

At the age of four, she presented with a urinary tract infection and was found to have marked bladder wall thickening and irregularity on ultrasound suggestive of neuropathic bladder, along with bilateral hydronephrosis. Functional studies demonstrated focal scarring of the right kidney. Urodynamics confirmed a large, atonic bladder with incomplete voiding. Chronic renal failure, with estimated function 50% of normal range first became evident aged seven. Due to anterior displacement of the bladder by progressive infiltrative lipomatous overgrowth, an obstructive uropathy component also developed, requiring placement of a suprapubic catheter.

By the age of thirteen, she was completely wheelchair bound and had frequent hospital admissions for recurrent urinary tract infections. Chronic pain became a severely disabling problem, and her progressive intra-abdominal overgrowth resulted in difficulties with lying and seating. She was treated with mTOR inhibitor sirolimus aged fifteen for a period of 12 months. Sub-therapeutic troughs were observed despite incremental dosing increases, and treatment was stopped after one year due to progression of her disease.

Progressive respiratory compromise from diaphragm elevation caused by intra-abdominal lipomatous overgrowth, and relentless flank pain from massive lipomatous distension determined the urgency of further treatment. Surgical therapeutic approaches were not favoured due to the infiltrative nature of fatty overgrowth encasing vital structures in the retroperitoneum, thus necessitating investigation of novel medical therapeutic approaches.

#### Patient two

**Fig. 1:**
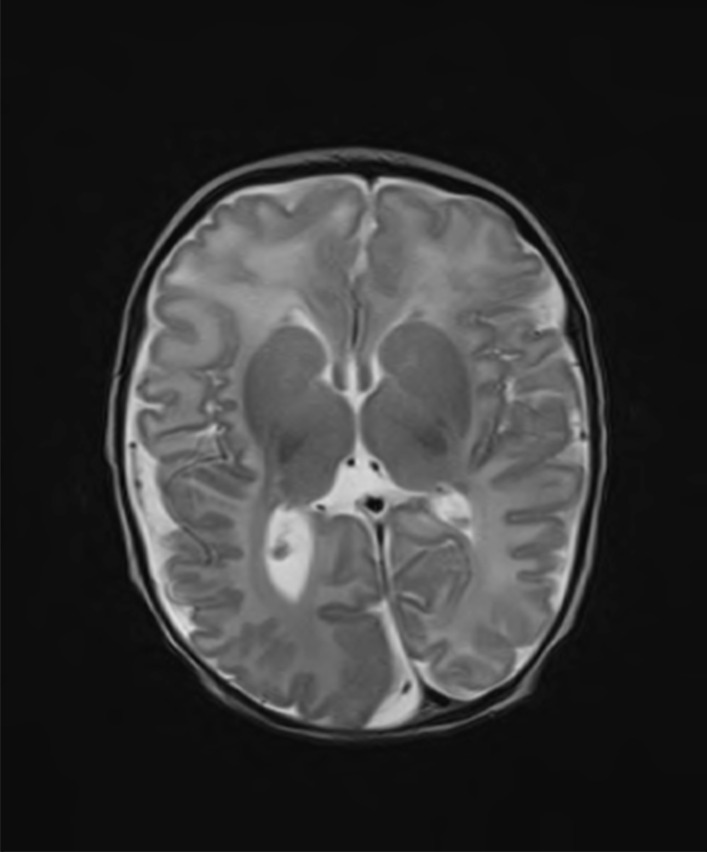
MRI at 2 weeks of age showing right hemimegalencephaly and polymicrogyria of the parietal lobe (patient two)

Patient two was a five years old male with PROS-associated facial infiltrating lipomatosis and hemimegalencephaly phenotype. He was born to non-consanguineous parents at term by elective Caesarian section for unstable lie with a birth weight of 4.15 kg (75th centile) and occipitofrontal circumference of 40.3 cm (> 99.6th centile). On examination he was noted to have right hemifacial macrosomia but no other signs of hemi-hypertrophy. His neonatal course was complicated by focal onset seizures day seven of life, characterised by right occipital discharges on electroencephalogram. Magnetic resonance imaging of the brain at three months reported right hemimegalencephaly and polymicrogyria of the parietal lobe with cortical thickening and malformation, particularly in the occipital lobe (Fig. 1).

**Fig. 2:**
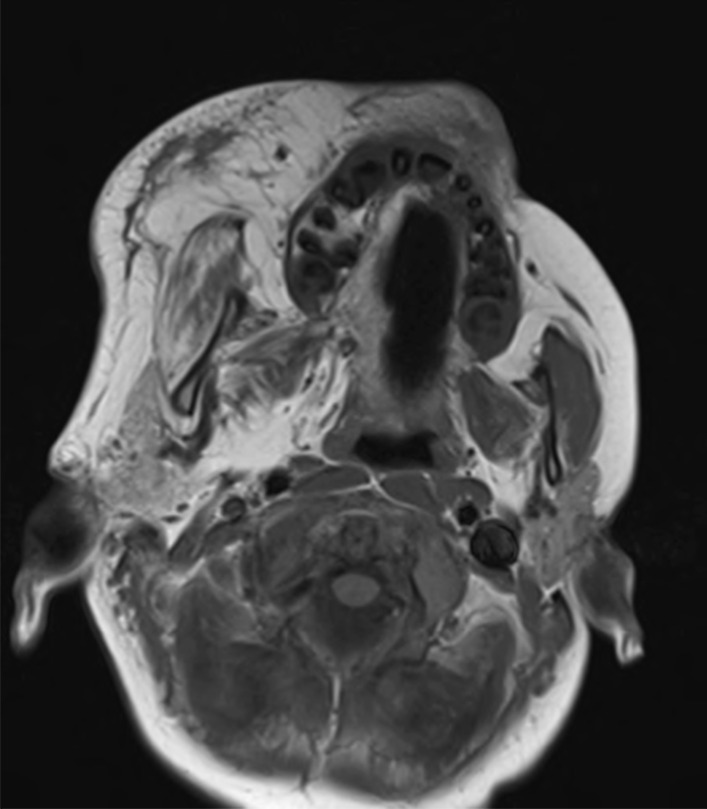
MRI at 4 years demonstrating extensive fatty infiltration and hypertrophy of the right side of the face (patient two)

During the first few years of life, profound hemifacial and soft tissue and skeletal overgrowth began to affect the oral commissure on the right side, and hemi-macroglossia and macrodontia caused progressive difficulties with articulation and oral function. Magnetic resonance imaging (MRI) showed extensive fatty hypertrophy and infiltration of the right side of the face, including the tongue and facial musculature (Fig. 2). Repeated liposuction and excision of excess tissue was undertaken for debulking and symmetrisation with a therapeutic aim of improving function. However progressive bony overgrowth was also evident which was deemed impossible to correct until skeletal maturation was reached. Executive functioning difficulties became apparent, consistent with right hemisphere abnormalities, reflected by attention and behavioural dysregulation difficulties. Additional educational support was also required due to low average scores obtained in general verbal and non-verbal skills on neuropsychological assessments.

He was commenced on sirolimus aged five for a period of eight months and achieved therapeutic levels of 5–10 ng/ml, but ongoing progression of his disease resulted in early discontinuation of treatment. Increased seizure frequency and an emerging subtle left-sided motor deficit in addition to relentless hemifacial overgrowth despite multiple surgical interventions rendered further consideration of targeted medical treatment important.

### Molecular investigations

Molecular investigations were undertaken in both patients to help guide therapeutic approaches. In patient one, studies of dermal fibroblasts cultured from an affected area revealed that levels the level of phosphorylation of AKT at serine 473, a downstream marker of PI3K, were 7 times higher than those in healthy controls, as determined by ELISA of a cellular lysate. Activation of the PI3K/AKT/MTOR pathway was assessed by assay of phosphorylation of AKT at residue p.Ser473 using a direct ELISA assay (InstantOne®, eBioscience). Early passage dermal fibroblasts cultured from were grown to 90% confluence, then serum starved for 24 h in DMEM containing 0.5% bovine serum albumin (Sigma). Cells were washed once in cold PBS, snap frozen in liquid nitrogen and stored at − 80 °C. Frozen monolayers were scraped in 150uL cell lysis buffer provided in the ELISA kit. Protein quantification was by DC Protein assay (BioRad) and lysates were diluted in lysis buffer to a protein concentration of 0.3–0.5 ng protein/µl before assay per manufacturer’s instructions. Briefly, 30–50 µg of cell lysate was loaded onto the pre-coated ELISA plate with 50 µl the primary and secondary antibody conjugates, and incubated for 1 h on a shaker. After washing, detection reagent was added, and was quenched with H2S04-containing stop solution. Absorbance was measured at 450 nm and results were normalized to the mean of five wild type control cell lines analysed at the same time. Analysis of patient cell ELISA scores (normalised to negative control cell scores) against *PIK3CA* genotype in a cohort of 118 people with segmental overgrowth who had undergone both cellular studies and deep genotyping of fibroblast DNA previously revealed a significant elevation of AKT-Ser473 phosphorylation in cells from patients with *PIK3CA* mutations versus cells from patients where no pathogenic *PIK3CA* mutation was found (p = 0.0098, Mann–Whitney U test). Using the arbitrary cut-off of a ‘positive’ ELISA score as more than two standard deviations above the mean of the negative control distribution, this test had the power to predict a *PIK3CA* mutation with a positive predictive value of 58% and a negative predictive value of 70% in our hands.

Subsequent genetic analysis of affected tissue identified a hotspot mutation in *PIK3CA* (c.3140A > G; p.His1047Arg) with reported variant frequency 12%. In patient two, genetic testing of affected fibroblasts demonstrated a different well established activating mutation (c.1258T > C; p.Cys420Arg) in *PIK3CA*, with variant frequency 15%, confirming PIK3CA-related overgrowth spectrum disorder. The mutations were revealed by targeted next generation sequencing (NGS) technology of genes involved in the P13K/AKT pathway and confirmed by Sanger sequencing. Next generation sequencing was carried out on Ion Torrent Personal Genome Machine (ThermoFisher Scientific, TFS), using the Ion PGM Sequencing Hi-Q 200 Kit (TFS) according to the manufacturer's instructions (Loconte et al. 2015). Data analysis was performed using the Torrent Suite Software v5.0.5 (TFS). Reads were aligned to the hg19 human reference genome from the UCSC Genome Browser (http://genome.ucsc.edu/) and to the BED file designed using Ion AmpliSeq Designer. Alignments were visually verified with the software Alamut® v2.8.0 (Interactive Bio software). Coverage and variant analysis were conducted according to methods previously reported by Loconte et al. [19].

### Investigational treatment with miransertib (ARQ 092)

Due to the severe, progressive nature of overgrowth in both cases resulting in increasing morbidity, and the lack of alternative available approved treatments, the use of novel experimental therapies was investigated. Applications for the compassionate use of the experimental drug, miransertib, were submitted to and approved by the Drugs and Therapeutics committee of Children’s Health Ireland, Crumlin (Dublin, Ireland); the Health Product Regulatory Agency of Ireland (HPRA) was informed of the orphan use of this drug and their adverse event reporting system was adhered to during the period of therapy. Initial dosing of 5 mg/m^2^/day was determined based on tolerability and efficacy reports in a phase 1 clinical trial investigating the use of miransertib in adult and paediatric patients over 12 years with PS [14]. ArQule Inc. (Burlington MA) provided miransertib on a managed patient access programme basis for both patients.

Informed consent was obtained from the legal guardians of both patients prior to treatment commencement. Baseline pre-treatment assessments were carried out, including evaluation of complete blood count, renal, liver, bone and fasting lipid profile and urinalysis. Transthoracic echo was undertaken to assess cardiac function. Volumetric MRI analysis of the targeted overgrowth area was also performed pre-treatment and every three months thereafter.

Subsequent monitoring included weekly assessment for glycosuria and point of care testing for hyperglycaemia. Evaluations of complete blood count, renal, liver and bone profile were repeated at week 4 and week 8 and every 8 weeks thereafter for the treatment duration. Adverse effects were classified according to the Common Terminology Criteria for Adverse Events, version 5.0 (CTCAE v.5.0) [19]. Where possible, serial volumetric analysis of DIXON MRI was carried out by a radiologist blinded to the patient’s medical history and visit schedule using Dynamika software (Image Analysis Group) as a platform for imaging data analysis.

## Results

### Patient one

Patient 1 commenced miransertib aged sixteen at a dose of 30 mg once daily and continued treatment for 28 months. Over the course of her treatment, an improvement in her respiratory compromise was observed, and functionally, she became able to lie flat. Her chronic renal failure was largely stable. Patient-reported quality of life measures improved.

**Fig. 3:**
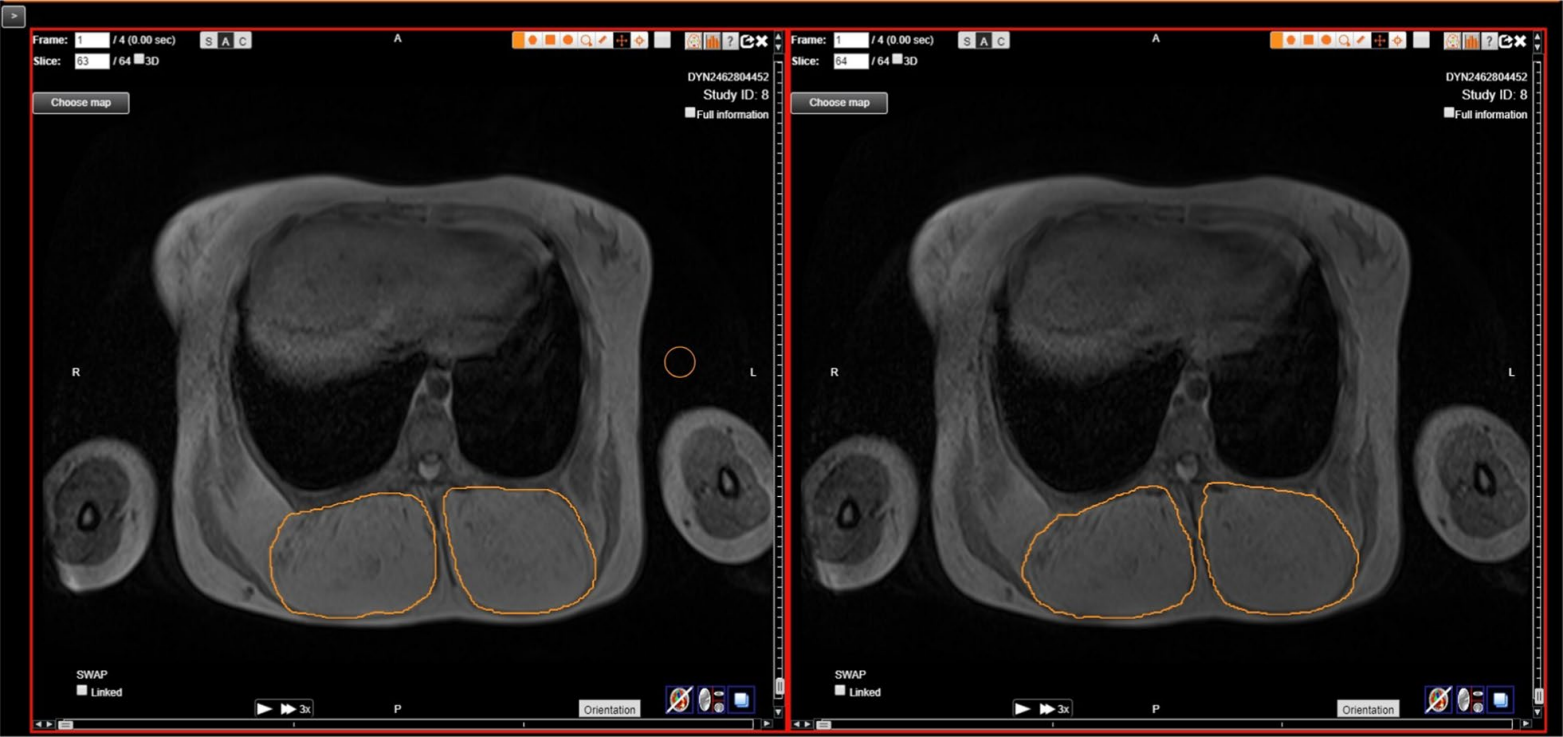
MRI of pelvis showing highlighted regions of interest (ROI) on adjacent slices, pre-treatment (left) and post 24 months treatment (right) (patient one)

Targeted regions of interest in MRI slices of the in-phase images were identified to define areas of fatty overgrowth. By treatment end, a 15% decline in the calculated volume of fatty overgrowth from baseline imaging was observed (Fig. 3).

Treatment with miransertib was well tolerated with no significant toxicities other than hyperlipidaemia, comparable to prior lipid profile derangement (grade 1 CTCAE v.5.0) with sirolimus therapy. Ultimately treatment was discontinued after 28 months due to poor patient adherence in the second year of therapy and failure of sustained beneficial effect, reflected by increased lipomatous volumes on MRI at 28 months.

### Patient two

Patient two commenced miransertib 10 mg once daily at 5 years of age, and continued treatment for 22 months. A subsequent reduction in seizure frequency was observed along with reported improved cognitive engagement in school and at home. Parental-reported quality of life measures, determined on clinical questioning at patient visits, improved. Treatment was very well tolerated with no adverse effects apart from loose stools (grade 1 CTCAE v.5.0), favourably comparable to that reported with previous sirolimus therapy. Objective assessment of response to treatment using volumetric MRI analysis was not possible due to ongoing surgical treatment of facial overgrowth during the treatment period.

Following discontinuation of miransertib treatment, both patients were commenced on targeted therapy with alpelisib (BYL719) based on the clinical efficacy in patients with PROS recently reported by Venot et al. [9]. Treatment is ongoing in both.

## Discussion

Here, we report the first paediatric case series of the use of miransertib in two children with severe PIK3CA-related overgrowth spectrum, one with CLOVES variant and one with facial infiltrating lipomatosis with hemimegalencephaly. The first patient demonstrated clear clinical improvement with observed stability in renal function and a 15% reduction in calculated volumes of fatty overgrowth at the targeted regions of interest. Functionally, improved lying and sitting postures were achieved, most importantly evidenced by the patient being able to lie flat again. These initial improvements were not sustained into the second year of therapy, largely due to poor treatment adherence, reflected objectively by increased lipomatous volumes on MRI at 28 months.

In the case of patient two, there was a significant improvement in parent- and patient-reported quality of life and function. Furthermore, a reduction in seizure burden on treatment was observed. Unfortunately, accurate volumetric analysis using MRI could not be used to monitor response to treatment due to ongoing surgical intervention. Due to the experimental nature of miransertib therapy, it was not deemed appropriate to defer any surgical treatments offered until response was evaluated. The use of miransertib did not preclude surgical treatment and was safe to use in the pre- and post-operative period. Similarly, serial photography was not a useful tool in either case, due to the targeted intra-abdominal site of interest in case one, and due to surgical treatment in case two. Quality of life improvements were self-reported in both cases. Quantitative assessment of quality of life using standardised tools such as Peds QL [20] is recommended in future studies.

The recently reported therapeutic utility of alpelisib (BYL719) in an open label case series (RCT data are awaited) of patients with PROS [9] demonstrates the potential availability of targeted therapies for patients with PROS. To date, we have observed a favourable safety profile in the sequential therapeutic use of miransertib and alpelisib in these two paediatric patients with severe PROS, highlighting the future potential for personalised medical treatment in these patients.

## Conclusion

The pan-AKT inhibitor miransertib (ARQ092) was well tolerated with no significant toxicities reported in two patients with severe PIK3CA-related overgrowth spectrum disorder. Treatment response and tolerability was superior to sirolimus in both cases. This case series highlights the potential therapeutic utility of miransertib in selected paediatric patients with severe PROS. An open label, Phase 1/2 study of miransertib administered to subjects at least 2 years of age with PROS and Proteus syndrome (PS) is currently underway to more accurately assess the efficacy of miransertib in the treatment of PIK3CA-related overgrowth spectrum disorder [21].

## Data Availability

Data sharing is not applicable to this article as no datasets were generated or analysed during the current study.
